# Gut microbiota therapy for nonalcoholic fatty liver disease: Evidence from randomized clinical trials

**DOI:** 10.3389/fmicb.2022.1004911

**Published:** 2023-01-16

**Authors:** Ting-Rui Han, Wen-Juan Yang, Qing-Hua Tan, Shuai Bai, Huang Zhong, Yang Tai, Huan Tong

**Affiliations:** ^1^West China School of Medicine, Sichuan University, Chengdu, China; ^2^Department of Gastroenterology, West China Hospital, Sichuan University, Chengdu, China; ^3^Lab of Gastroenterology and Hepatology, West China Hospital, Sichuan University, Chengdu, China; ^4^Department of Gastroenterology, Zigong First People’s Hospital, Zigong, China

**Keywords:** non-alcoholic fatty liver disease, gut microbiota, randomized clinical trial, probiotics, prebiotics, synbiotics, fecal microbiota transplantation, antibiotics

## Abstract

Nonalcoholic fatty liver disease (NAFLD) has a high prevalence worldwide, but there are no medications approved for treatment. Gut microbiota would be a novel and promising therapeutic target based on the concept of the gut–liver axis in liver disease. We reviewed randomized controlled trials on gut microbiota therapy in NAFLD in this study to evaluate its efficacy and plausibility in NAFLD.

## Introduction

The high global prevalence of nonalcoholic fatty liver disease (NAFLD; 25.2%) renders this disease a public health concern worldwide (Younossi et al., 2016). Deteriorating hepatic steatosis triggers persistent inflammation in the liver and, thereafter, inflicts fibrosis/cirrhosis or even hepatocellular carcinoma. Although lifestyle modification is the cornerstone of the treatment for NAFLD, only 10% succeed in losing ≥10% of the body weight, which is considered effective to contain steatosis, fibrosis, and nonalcoholic steatohepatitis (Alkhouri and Scott, 2018). Therefore, there is still an urgent need for medications to combat NAFLD. In the past decade, medications for treating NAFLD have constantly emerged in various categories, and those medications target the peroxisome proliferator-activated receptor, the farnesoid X nuclear receptor, glucagon-like peptides, etc. (Dufour et al., 2022). However, most of them have been investigated in randomized clinical trials (RCTs), and they are not yet licensed for routine clinical use.

The gut, where trillions of bacteria reside, has come under the spotlight for novel understandings and innovative treatments for many diseases in recent years. An increasing body of evidence suggests a link among the gut and its microbiota and NAFLD, which is termed the gut–liver axis (Albillos et al., 2020). Bile acid signaling, microbial metabolism, and bacterial translocation jointly constitute the key pathways of the gut–liver axis and its axis disruption was recently accepted as one pathogenesis of NAFLD (Bauer et al., 2022). As an indispensable component of the gut–liver axis, the gut microbiota has become a potential therapeutic target for NAFLD. Herein, we reviewed RCTs indexed in PubMed for the gut microbiota therapy of NAFLD. The baseline characteristics and main clinical outcomes are summarized in Tables 1, 2.

**Table 1:** Baseline characteristics of randomized clinical trials of gut microbiota therapy in nonalcoholic fatty liver disease.

Authors	Country	Fatty liver diagnosing method	Sample size (control vs. treatment)	Age (years, control vs. treatment)	Gender (men/women, control vs. treatment)	BMI (kg/m^2^, control vs. treatment)	Diabetes (%, control vs. treatment)
Alisi et al. (2014)^a^	United States	Biopsy	22 vs. 22	11 vs. 10	14/8 vs. 10/12	25.6 vs. 27.3	No data
Kobyliak et al. (2018)	Ukraine	Ultrasonography	28 vs. 30	57.3 vs. 53.4	No data	34.26 vs. 34.82	100% vs. 100%
Anh et al. (2019)	Korea	MRI-PDFF	35 vs. 30	44.7 vs. 41.7	18/18 vs. 15/17	30.11 vs. 30.05	No data
Sepideh et al. (2016)	Iran	Ultrasonography	21 vs. 21	47.3 vs. 42.1	15/6 vs. 13/8	29.50 vs. 30.34	No data
Bomhof et al. (2019)	Canada	Biopsy	6 vs. 8	53.3 vs. 45.3	3/3 vs. 5/3	34.8 vs. 33.7	No data
Behrouz et al. (2020)^b^	Iran	Ultrasonography	30 vs. 30 vs. 29	38.4 vs. 38.5 vs. 38.4	21/9 vs. 22/8 vs. 20/9	30.12 vs. 29.05 vs. 29.32	0% vs. 0% vs. 0%
Malaguarnera et al. (2012)	Italy	Ultrasonography	32 vs. 34	46.7 vs. 46.9	15/17 vs. 18/16	27.2 vs. 27.3	15.6% vs. 17.6%
Ferolla et al. (2016)	Brazil	Biopsy	23 vs. 27	57.3 for all patients	12/38 for all patients	32.5 vs. 32.5	45.5% vs. 32.1%
Shavakhi et al. (2013)	Iran	Biopsy	32 vs. 31	38.7 vs. 41.5	17/14 vs. 15/17	28.2 vs. 28.6	No data
Chen et al. (2019)	China	Ultrasonography	44 vs. 48	51.2 vs. 48.9	0/44 vs. 0/48	31.81 vs. 32.18	0% vs. 0%
Scorletti et al. (2020)^a^	Great Britain	Radiology or biopsy	44 vs. 45	51.6 vs. 50.2	61/39 vs. 69/31	33.2 vs. 32.9	36% vs. 38%
Craven et al. (2020)	Canada	Not clarified	6 vs. 15	57.5 vs. 47.6	1/5 vs. 5/10	37.4 vs. 36.3	0% vs. 0%
Chong et al. (2020)^c^	New Zealand	Biopsy	20 vs. 20 vs. 20	46.7 vs. 51.5 vs. 50.6	13/7 vs. 8/12 vs. 10/10	32.6 vs. 30.9 vs. 31.4	35% vs. 35% vs. 35%

a Quantitative data in these rows are described as medians. Quantitative data in other rows are described as means.

b Data are presented as control vs. probiotics vs. prebiotics.

c Data are presented as placebo–placebo vs. placebo–inulin vs. metronidazole–inulin.

**Table 2:** Primary outcomes, liver evaluation method, and main positive outcomes of randomized clinical trials of gut microbiota therapy in nonalcoholic fatty liver disease.

Authors	Treatment & duration	Primary outcomes	Liver evaluation method	Main positive outcomes of microbiota therapy
Alisi et al. (2014)	Probiotics (4 months)	Fatty liver severity	Ultrasonography	Less severe hepatic steatosis, lower BMI, higher blood GLP-1, and α-GLP-1
Kobyliak et al. (2018)	Probiotics (8 weeks)	FLI and liver stiffness	FLI and Shear Wave Elastography	More decreased FLI, serum AST, gamma-glutamyl transpeptidase, TNFα, and IL6
Anh et al. (2019)	Probiotics, 12 weeks	VFA and IHF	MR	More decreased IHF and body weight
Sepideh et al. (2016)	Probiotics (8 weeks)	Not clarified	Not evaluated	Lower fasting blood sugar, insulin, HOMA-IR, and IL6
Bomhof et al. (2019)	Prebiotics (36 weeks)	NAS score	Liver biopsy	Improved hepatic steatosis and NAS score
Behrouz et al. (2020)	Probiotics (12 weeks), prebiotics (12 weeks)	Not clarified	Not evaluated	Decreased serum leptin, insulin, and HOMA-IR in the probiotic and prebiotic groups and decreased fasting blood sugar only in the prebiotic group
Malaguarnera et al. (2012)	Synbiotics (24 weeks)	NASH activity index	Liver biopsy	Lower AST, low-density lipoprotein, C-reactive protein, TNFα, and HOMA-IR; better mitigated hepatic steatosis and NASH activity index
Ferolla et al. (2016)	Synbiotics (12 weeks)	Not clarified	MR	Reduction in liver steatosis, body weight, BMI, and waist circumstance
Shavakhi et al. (2013)	Synbiotics (6 months)	ALT, AST, and ultrasound grade of NASH	Ultrasound	Lower ALT, AST, BMI, blood triglyceride, and cholesterol. Lower grade of hepatic steatosis
Chen et al. (2019)	Synbiotics (24 weeks)	Not clarified	MR	Lower waist circumstance, HOMA-IR, hepatic fat fraction, intraheaptic lipid, serum LPS, and FGF21; higher GSH-Px and SOD
Scorletti et al. (2020)	Synbiotics (24 weeks)	Liver fat fraction, liver fibrosis score, and gut microbiota composition	MR	Altered fecal microbiota composition
Craven et al. (2020)	Fecal microbiota transplantation (6 months)	Insulin resistance	MR	Reduced small intestinal permeability without improved HOMA-IR and hepatic proton density fat fraction
Chong et al. (2020)	Prebiotics (12 weeks) + antibiotics (1 week)	≥7% weight loss	Transient elastography	Lower ALT and AST

ALT: alanine aminotransferase; AST: aspartate aminotransferase; BMI: body mass index; FGF21: fibroblast growth factor 21; FLI: fatty liver index; GGT: γ-glutamyl transferase; GLP: glucagon-like peptide; GSH-Px: glutathione peroxidase; HOMA-IR: homeostasis model assessment-insulin resistance; IHF: intrahepatic fat; IL6: interleukin 6; LPS: lipopolysaccharide; MR: magnetic resonance; NAS: non-alcoholic fatty liver disease activity score; NASH: non-alcoholic steatohepatitis; SOD: superoxide dismutase; TNFα: tumor necrosis factor α; VFA: visceral fat area.

To review this topic, references indexed in PubMed (published from the year of 2001 to 2021) were searched with the keywords of (“probiotics,” “randomized clinical trial,” and “non-alcoholic fatty liver disease”), (“prebiotics,” “randomized clinical trial,” and “non-alcoholic fatty liver disease”), (“synbiotics,” “randomized clinical trial,” and “non-alcoholic fatty liver disease”), (“fecal microbiota transplantation,” “randomized clinical trial,” and “non-alcoholic fatty liver disease”), and (“antibiotics,” “randomized clinical trial,” and “non-alcoholic fatty liver disease”). A total of 96 references were initially identified, and finally, 13 published RCTs were included in this mini-review.

### Probiotics

Probiotics have been defined by the International Scientific Association for Probiotics and Prebiotics (ISAPP) as “live microorganisms that, when administered in adequate amounts, confer a health benefit on the host” (Hill et al., 2014). It has become increasingly popular for medicine and food industries to study and promote probiotic bacteria in recent years because of their therapeutic benefits for various diseases, such as inflammatory bowel disease, allergy, and diarrhea (Terpou et al., 2019). In addition, accumulating evidence showed that intestinal bacterial changes affect liver lipid metabolism, and probiotics can treat liver disease (Albillos et al., 2020).

An RCT conducted by Alisi et al. (2014) included 44 obese subjects with biopsy-proven NAFLD to investigate the efficacy of VSL3# in NAFLD. The trial was a double-blind study with a duration of 4 months. VSL3# is a mixture of eight probiotic strains, which contains *Streptococcus thermophilus*, bifidobacteria (*Bifidobacterium breve*, *Bifidobacterium infantis*, and *Bifidobacterium longum*), *Lactobacillus acidophilus*, *Lactobacillus plantarum*, *L. paracasei*, and *Lactobacillus delbrueckii* subsp. *bulgaricus*. In this trial, the subjects in the VSL3# group received one or two sachets depending on whether the child was older than 10 years. As a result, the children supplemented with VSL3# bore only 9% moderate-to-severe liver steatosis under liver pathology as compared to 93% of those receiving placebo. In addition, VSL3# conferred a higher BMI decrease (VSL3# vs. placebo −2.2 vs. 0.1 kg/m^2^), especially the baseline BMI in the VSL3# group, which was higher than that in the placebo group, making the weight loss more impressive under VSL3#. In addition, greater increases in blood GLP-1 and a-GLP-1 were found in the VSL3# group, which benefited metabolism. However, the blood alanine aminotransferase, triglyceride, and HOMA-IR levels did not improve after 4 months of VSL3# administration. Although VSL#3 seems to play a beneficial role in alleviating NAFLD, it is noteworthy that the conclusion of the effect of VSL3# on NAFLD could not be simply applied to adults as children were the unique population enrolled in this study. In addition, hepatocyte inflammation and ballooning and fibrosis in liver specimens were not properly studied after treatment.

In another RCT conducted by Kobyliak et al. (2018), 58 adult NAFLD participants with type-2 diabetes received the multiprobiotic “Symbiter” 1 sachet per day, which consists of the combination of 14 probiotic bacteria genera (*Bifidobacterium*, *Lactobacillus*, *Lactococcus,* and *Propionibacterium*) or placebo. The trial was double-blinded with a duration of 8 weeks. The probiotic group conferred lower levels of serum AST and γ-GT after the intervention, while no significant change was found in the ALT level or the lipid profile. Meanwhile, TNF-α and IL-6 decreased under the administration of Symbiter. Fatty liver index (FLI) and liver stiffness (LS) were used to evaluate the liver histology shift, indicating that only FLI was improved with a decrease of 5.6 after Symbiter was given, and LS was not mitigated significantly by Symbiter. However, liver pathology was not employed to obtain a better assessment, although this study favored probiotics in adult NAFLD with type-2 diabetes. In addition, the small sample size and short follow-up (8 weeks) might undermine the rigor of the results.

The effect of probiotics on NAFLD was also assessed in an Asian population in a 12-week, double-blind, randomized clinical trial (Anh et al., 2019). A total of 65 adults from Korea with NAFLD were included. The probiotic mixture comprised six bacterial species (*L. acidophilus*, *Lactobacillus rhamnosus*, *L. paracasei*, *Pediococcus pentosaceus*, *Bifidobacterium lactis*, and *B. breve*). The blood cholesterol, triglyceride, HDL, and TNF-α levels were markedly lower in the probiotic group than in the control group, whereas the glucose, insulin, ALT, and AST levels were not significantly changed. Moreover, in addition to body weight and total body fat reductions, MRI-PDFF showed that the intrahepatic fat fraction decreased after probiotics were prescribed for 12 weeks. The probiotic group had 22.9% more improvement and 18.1% less worsening in intrahepatic fat fraction than the control group. However, 12 weeks of treatment is a relatively short duration to evaluate the efficacy of a treatment for chronic diseases such as NAFLD, and the discordance of different imaging methods (MR-PDFF and CAP) in evaluating hepatic steatosis compromised the accuracy of the conclusions.

One double-blind RCT tested the glycemic and inflammatory indices in NAFLD among Iranian populations (Sepideh et al., 2016). The probiotic Lactocare is composed of seven strains of bacteria (*Lactobacillus casei*, *L. acidophilus*, *L. rhamnosus*, *Lactobacillus bulgaricus*, *B. breve*, *B. longum*, and *S. thermophilus*). A total of 21 participants were enrolled in each arm. After the 2-month intervention, fasting blood sugar, insulin, and HOMA-IR in the probiotic group decreased compared to their baselines, whereas in the placebo group, these variables increased from their baselines (fasting blood sugar change: probiotic vs. placebo −4.57 vs. 2.62 mmol/L; insulin: probiotic vs. placebo −2.52 vs. 1.82 μU/mL; and HOMA-IR: probiotic vs. placebo −0.51 vs. 0.33). Regarding inflammatory parameters, TNFα and IL6 were mitigated in the probiotic group but deteriorated in the placebo group. Although glucose metabolism, insulin resistance, and inflammation were improved in the wake of probiotic administration, any change in the liver histology was not specifically studied in this RCT. Regardless, as insulin resistance is fundamental to NAFLD development, the outcome that insulin resistance was improved in this RCT still sheds light on the probiotic treatment of NAFLD.

The published RCTs on probiotics treating NAFLD shared some common disadvantages. Most of them did not stringently evaluate liver histology, and the liver change after probiotic treatment was not fully investigated. Follow-ups were not long enough to properly evaluate the clinical efficacy. In addition, the bacterial species in each RCT differed, and it is difficult to conclude whether probiotics benefit NAFLD.

### Prebiotics

Over 20 years ago, a class of compounds, termed prebiotics, were recognized for their ability to manipulate host microbiota to the benefit of the host (Gibson and Roberfroid, 1995). Currently, the concept of prebiotics has been expanded. The ISAPP consensus statement redefines prebiotics as “a substrate that is selectively utilized by host microorganisms conferring a health benefit” (Gibson et al., 2017). Prebiotics comprise one or more indigestible carbohydrates that escape digestion in the small intestine but are metabolized by microbes in the colon, where they are fermented into short-chain fatty acids (SCFAs), gases, and other products (Scott et al., 2013). These metabolites improve the intestinal environment as a nutrient source to help probiotic growth, affecting host health (Markowiak and Slizewska, 2017). The commonly used prebiotics include fructooligosaccharide (FOS), inulin, and guar gum.

Bomhof et al. (2019) conducted an RCT including 14 patients with biopsy-proven NASH from Canada. The subjects in the prebiotic group took FOS for 36 weeks Compared to the placebo group, the prebiotic group did not have any improvement in body weight or body fat, but it had better hepatic steatosis and NAS score mitigations, as well as lower fasting glucose. However, two issues should be noted in this study. First, the sample size was small in both the prebiotic and placebo groups. Bias in the conclusion probably existed because fewer than 10 subjects were enrolled in both groups. Second, in addition to hepatic steatosis, lobular inflammation and ballooning are two other important traits of liver pathology. In this study, only steatosis was alleviated, but neither lobular inflammation nor ballooning was mitigated. This finding could be attributed to either the fact that prebiotics could only have an impact on hepatic steatosis or the fact that the administration duration of prebiotics was not long enough to affect lobular inflammation and ballooning, and further studies are needed to clarify the issue.

Behrouz et al. (2020) investigated the effects of prebiotics and probiotics in 89 Iranian patients with NAFLD. In this 12-week RCT, the prebiotic group received FOS at a dosage of 16 g per day, whereas the probiotic group received one capsule daily containing 5 billion of five bacterial strains (*Lactobacillus casei*, *L. rhamnosus*, *L. acidophilus*, *B. longum*, and *B. breve*). Both body weight and body mass index (BMI) decreased in all three groups after 12 weeks of treatment, but neither prebiotics nor probiotics showed an advantage in such a decrease over placebo. Compared with the control group, both the probiotic and prebiotic groups showed improved leptin, adiponectin, insulin, and HOMA-IR after treatment. Moreover, prebiotics showed an extra advantage over probiotics in mitigating fasting blood glucose. Although the circulating metabolism variables were improved by probiotics and prebiotics, no information on liver histology was obtained in this study as liver biopsy was not performed.

Therefore, based on the above review, although prebiotics were found to mitigate metabolic variables in NAFLD individuals, however, high-quality RCTs are still warranted in the future due to the limited number and the insufficient strength of RCTs conducted.

### Synbiotics

In formulating this concept, synbiotics were loosely described as mixtures of “probiotics and prebiotics that beneficially affect the host” (Gibson and Roberfroid, 1995). Unfortunately, this definition is too broad and lacks precision. ISAPP recently reviewed the definition and scope of synbiotics and updated its definition as “a mixture comprising live microorganisms and substrate(s) selectively utilized by host microorganisms that confers a health benefit on the host” (Swanson et al., 2020). Synbiotics incorporate prebiotic and probiotic components and are created to promote the survival and colonization of live microbes in the gastrointestinal tract (Neish, 2009). Therefore, an appropriate combination of both components in a single product may produce a superior effect compared to either component alone (Verma et al., 2012).

Malaguarnera et al. (2012) carried out a 24-week RCT in Italy including 66 patients with biopsy-proven NASH. The synbiotics given in this study were a combination of *B. longum* and FOS. The results showed that, compared with the placebo, the synbiotics exerted extra ameliorations on serum inflammation parameters, such as TNFα, endotoxin, CRP, and AST, and had little impact on serum metabolism parameters except for LDL and HOMA-IR. Moreover, regarding liver histology, the synbiotics only improved hepatic steatosis and did not have an additional effect on hepatic inflammation and fibrosis when compared with the placebo. Therefore, the combination of *B. longum* and FOS seems to have a mild therapeutic effect on NASH.

Ferolla et al. (2016) studied the effect of synbiotic supplementation on NASH. This RCT enrolled 50 patients with biopsy-proven NASH. The synbiotics in this study contained 10^8^ CFU of *Lactobacillus reuteri* plus 4 g of partially hydrolyzed guar gum and inulin, and they were given two times daily to those in the synbiotic group. After 12 weeks of treatment, synbiotics decreased body weight, BMI, and waist circumference, whereas the liver biochemistry profile did not undergo an apparent shift. Although the synbiotics did not downregulate the lipid profile level, triglycerides and VLDL in the control group were elevated at the end of the study. Meanwhile, the hepatic proton density fat fraction decreased by 22.8% in the synbiotic group in contrast to the 14.1% increase found in the control group. However, biopsy data that present a thorough evaluation of liver changes induced by synbiotics were not collected.

Another double-blind RCT conducted in Iran investigated the role of probiotics, *de facto* synbiotics, in NAFLD by comparing the regimens of metformin and metformin plus synbiotics (Shavakhi et al., 2013). The synbiotics in this RCT contained *Lactobacillus acidophilus*, *L. casei*, *L. rhamnosus*, *L. bulgaricus*, *B. breve*, *B. longum*, *Streptococcus thermophilus*, and FOS. After 6 months of treatment in 63 patients, the supplementation of synbiotics conferred participants with more decrements of ALT, AST, blood triglyceride, cholesterol, and BMI, compared with a single use of metformin. BMI, another impressive variable, was 4.8 kg/m^2^ less in patients prescribed metformin plus synbiotics after 6 months of treatment. In addition, after 6 months of treatment with synbiotics, less grade 2–3 hepatic steatosis was found under ultrasound (metformin+placebo: 46.9% and metformin+synbiotics: 0%). However, the investigators did not determine the severity of hepatic steatosis with biopsy, MRI-PDFF, or even CAP by ultrasound; therefore, the accuracy of the evaluation of the change in the liver is debatable.

Yogurt is a kind of fermented food abundant in both probiotics and prebiotics, which are beneficial for human health. Chen et al. (2019) investigated how yogurt affected NAFLD and metabolic syndrome in 92 Chinese individuals with obesity, 48 of whom consumed 220 g of yogurt daily for 24 weeks. It was impressive to find that yogurt consumption conferred participants with 2.24 kg more fat mass decrease and 1.85 cm more waist circumference decrease than the controls after 24 weeks. Participants consuming yogurt had a decrease in BMI by −0.28 kg/m^2^ than those consuming milk, but this difference did not reach significance. Serum sugar and lipid profiles were lower after 24 weeks of yogurt intake, in addition to better insulin resistance alleviation (adjusted HOMA-IR yogurt vs. milk: −0.53). Moreover, the hepatic fat fraction and intrahepatic lipids were 3.48 and 3.44% lower in yogurt-consuming individuals, respectively. Yogurt was also found to mitigate inflammation and oxidative stress and alter gut microbiota composition in this study. Notably, this trial only enrolled women, and the microbiota variables are difficult to unify due to the different manufacturing protocols. Yogurt is a more compliable and acceptable therapy over medications.

From the aforementioned studies, it seems that synbiotics play a positive role in NAFLD; however, Scorletti et al. (2020) provided an opposite description in a double-blind RCT including 89 British patients. The synbiotics in this study consisted of FOS and *Bifidobacterium animalis* subsp. lactis BB-12. After 10–14 months of intervention, although the fecal samples of the synbiotics group had higher proportions of *Bifidobacterium* and *Faecalibacterium* and reductions in *Oscillibacter* and *Alistipes*, the synbiotics did not improve the body weight, BMI, serum glycemic or lipid parameters. More importantly, the synbiotics did not alter liver fat or liver fibrosis, casting a shadow over synbiotics in NAFLD. An advantage of this RCT lies in the record of physical activity in METs, which was neglected by the RCTs on NAFLD, therefore introducing bias to the clinical efficacy of medications.

### Fecal microbiota transplantation

Fecal microbiota transplantation (FMT), also known as stool transplantation, is the transplantation of fecal microbiota suspension from a healthy donor to a diseased recipient in an attempt to restore microbiota diversity and composition (Gupta and Khanna, 2017; Khoruts, 2018). Since the approval of FMT for the treatment of recurrent *Clostridium difficile* infection in 2014 (Mullish et al., 2018). FMT has shown promising results in clinical trials of a variety of gastrointestinal disorders resulting from gut dysbiosis beyond *C. difficile* infection, such as irritable bowel syndrome (IBS) and inflammatory bowel disease (Borody and Khoruts, 2012). It has long been known that the dysbiosis of the intestinal microbiota may be an underlying mechanism that leads to metabolic disorders, such as obesity, diabetes, and fatty liver (Zhao, 2013).

A 6-month double-blind RCT conducted by Craven et al. (2020) suggested that allogenic FMT could reduce the lactulose/mannitol ratio; therefore, intestinal permeability was indicated to improve, which was speculated to prevent insulin resistance and NAFLD in individuals. However, no significant changes were shown in either hepatic PDFF or HOMA-IR. Although the outcomes discourage FMT in NAFLD treatment, there are still some disadvantages that might downplay the FMT effect. In addition to the small sample size in each group (*n* = 21 in total), the arrangement of FMT might be inappropriate. The investigators only deployed FMT one time, but the follow-up period assigned was 6 months to detect its efficacy. It would be difficult to expect one session of FMT to exert and maintain a long-lasting therapeutic effect on the liver *via* the gut. Thus, future studies including repeated FMT sessions in more patients with NAFLD with a long follow-up are still needed.

### Antibiotics

Antibiotics act as double-edged swords in the gut microbiota. On the one hand, antibiotics could disturb the microbiota profile of the gut, and unreasonable administration of antibiotics elicits clinical problems such as *C. difficile* infection. On the other hand, the subtle application of antibiotics sheds a light on tough clinical situations. For example, rifaximin alters the gut microbiota components, improves the clinical outcomes of hepatic encephalopathy *via* the gut–liver axis, and therefore becomes the first-line medication for hepatic encephalopathy (Vilstrup et al., 2014).

New Zealand researchers investigated metronidazole with inulin in NAFLD with elevated transaminase (Chong et al., 2020). The participants received a complex regimen in which individuals received metronidazole (400 mg two times daily for 7 days) along with inulin (4 g two times daily for 12 weeks) in the treatment group (MI, *n* = 20), whereas participants in the control groups either received metronidazole-like placebo and inulin (IP, *n* = 20) or placebo only (PP, *n* = 20). When the data were retrieved at 16 weeks, although ALT and AST underwent a notable decrease in MI (ALT: −19.6 U/L, AST: −14 U/L), the MI regimen did not show enough efficacy in alleviating blood weight, blood glucose, blood lipids, or CAP.

Regarding antibiotic treatment for NAFLD, data are limited due to the concerns that improper antibiotic administration might lead to the potential sequelae of gut dysbiosis. Nevertheless, antibiotics includes various kinds of medications with different priority targets on the bacterial strains in the gut, making it challenging to obtain a sweeping conclusion on whether an antibiotic is beneficial or deteriorates to NAFLD.

### Future perspective

The huge bank of bacteria in the gut, together with the gut–liver axis, renders the microbiota a potential treatment target for NAFLD. However, as the profile of gut microbiota is subject to various factors, including geographical distribution, diet, medications, environmental factors, and development stage, significant heterogeneity lies in gut microbiota between individuals (Anwar et al., 2021). It would be better not to liberally apply the conclusions of the aforementioned RCTs to another population, which casts a shadow on the practice of this treatment strategy. A deep understanding of the gut microbiota profile and its modulatory factors would pave the way to resolve this issue.

On the contrary, lifestyle changes, an overlooked issue in NAFLD RCTs, might pose bias in investigating medical efficacy, including microbiota therapy. Although recommendations for the standardization of diet and exercise in clinical trials of NAFLD-NASH were published in 2020 (Glass et al., 2020), the lifestyle changes referring to diet and exercise are intractable to quantify accurately. Therefore, new evaluation tools are needed to minimize the interference of life change with RCTs of the gut microbiota therapy in NAFLD and would be helpful to obtain a better understanding of gut microbiota therapy in NAFLD.

Although current outcomes on gut microbiota therapy of NAFLD vary between RCTs, some aforementioned RCTs still demonstrate that NAFLDs might benefit from gut microbiota therapy due to insulin resistance improvement, steatosis mitigation, hepatic inflammation, and fibrosis alleviation. While there are a series of obstacles with gut microbiota therapy, it is still expected to be promising in NAFLD treatment.

## Author contributions

HT and YT contributed to conception and design of the study. T-RH and YT wrote the first draft of the manuscript. W-JY, Q-HT, SB and HZ wrote sections of the manuscript. HT reviewed and revised the manuscript. All authors contributed to the article and approved the submitted version.

## Funding

This study was supported by the National Natural Science Fund of China (81700539), the Sichuan Science and Technology Program (2020YFS0236), and the Corporation project of Sichuan University and Zigong (2022CDZG-26).

## Conflict of interest

The authors declare that the research was conducted in the absence of any commercial or financial relationships that could be construed as a potential conflict of interest.

## Publisher’s note

All claims expressed in this article are solely those of the authors and do not necessarily represent those of their affiliated organizations, or those of the publisher, the editors and the reviewers. Any product that may be evaluated in this article, or claim that may be made by its manufacturer, is not guaranteed or endorsed by the publisher.
